# Complex Activity and Sensor Potential toward Metal Ions in Environmental Water Samples of N-Phthalimide Azo-Azomethine Dyes

**DOI:** 10.3390/molecules26195885

**Published:** 2021-09-28

**Authors:** Stela Georgieva, Artem Bezfamilnyi, Anton Georgiev, Marian Varbanov

**Affiliations:** 1 Department of Analytical Chemistry, University of Chemical Technology and Metallurgy, 1756 Sofia, Bulgaria; bezfamilnyiartem@gmail.com; 2 Department of Organic Chemistry, University of Chemical Technology and Metallurgy, 1756 Sofia, Bulgaria; antonchem@uctm.edu; 3 Institute of Optical Materials and Technologies, Bulgarian Academy of Science, Acad. G. Bonchev Avenue, bldg. 109, 1113 Sofia, Bulgaria; 4 Department of Geography, National Institute of Geophysics, Geodesy and Geography, Bulgarian Academy of Sciences (NIGGG-BAS), Str. “Acad. G. Bonchev”, bl.3, 1113 Sofia, Bulgaria; marian.varbanov@gmail.com

**Keywords:** azo-azomethine dyes, sensors, metal ion, indicator, titration, electrochemistry, spectrometry

## Abstract

Herein, the spectral and electrochemical characterizations of three different substituted N-phthalimide azo-azomethine (NAA) dyes (L) containing an o-hydroxy group and their NAA-M(II) chelates [M(II): Cu, Ni, Co, Pb] were reported by using UV–Vis and fluorescence spectroscopy and potentiometric and voltamperometric techniques. The pK value of the dyes as well as the stoichiometry and stability of the NAA-metal chelates were studied, and the stoichiometry was found to be mostly 1:2 (ML_2_) with high complex stability constant values. The sensor activity of N-phthalimide azo-azomethine derivatives toward pH and metal ions has been also investigated and tested for indicator application in acid–base analysis and detection of Cu(II) ions in real samples of surface river water using voltamperometric detection. The results showed that one of the ligands possesses the highest electrochemical response upon binding to copper ions and could be successfully used in the analysis of copper in water at a concentration range of the analyte from 3.7 × 10^−7^ to 5.0 × 10^−6^ mol L^−1^, with analytical characteristics of the method being Sr = 1.5%, LOD = 3.58 µg L^−1^ and LOQ =11.9 µg L^−1^

## 1. Introduction

The development and study of chemosensors is an area of analysis that receives considerable attention due to their direct ability to recognize heavy metal ions in key areas such as clinics, biochemistry, and the environment [1]. Thus, the development of effective molecular sensors for the detection of metal ions is an evolving field in chemistry due to their simplicity, low cost, sensitive ion-induced spectroscopic and electrochemical changes, easy monitoring of operating conditions, and analysis in real time [2,3,4]. Many chemists have focused on the advances in the analysis of azo dyes, which has led to their direct use as chromophoric and metallochromic reagents [5,6], high-performance materials [7,8], photo-sensitizers [9,10], colorants [11,12,13], environmental sensors for substrate detection [14,15,16] and analytical reagents; they are important in the production of paints because they cover a large range of colors (practically every shade from yellow to black) [17,18]. Azo multifunctional dyes, in addition to the characteristic azo (-N=N-) group [19], could contain various substituents in aromatic rings or groups involved in complex formation processes, such as azomethine (-CH=N-), that can enhance or hinder their application [20,21,22,23]. On the other hand, the combination of the presence of azo and azomethine groups in the structure of the compound results in obtaining an important class of organic compounds that combine the properties to change color in a particular medium, depending primarily on the azo group, and to coordinate with metal ions through the azo or azomethine part from a molecule [24]. Thus, azo-azomethine derivatives have become potential sensors for trapping metal ions as they readily form reasonably stable complexes with most transition metal ions. Compared to the dyes themselves, metal dyes are more light-stable and have good thermal stability and physicochemical characteristics, which facilitates the use of azo and azo-azomethine compounds, for example, in optical data storage and as metal environmental sensors [25,26].

The problem of environmental pollution has been a priority topic in environmental research in recent years, as all habitats of organisms—soil, water, and air—are polluted in one way or another, and this has negative consequences for flora and fauna [27,28,29,30,31]. Many different substances are considered pollutants in the environment, ranging from simple inorganic ions to complex organic molecules. In general, metals are considered as such due to their toxicity, resistance, and tendency to accumulate both in the environment and in the human body, especially during chronic exposure [28]. However, in most cases they become pollutants when, in anthropogenic activities, mainly mining (ore mining and smelting; industry and technology), they are released from the rocks that contain them and in the process can cause damage to nature [29,30]. Therefore, the modern development of human activities requires the use of highly sensitive methods of analysis, for example, of metals or metal forms that allow the detection of micro and nano quantities of various substances.

**Figure 1:**
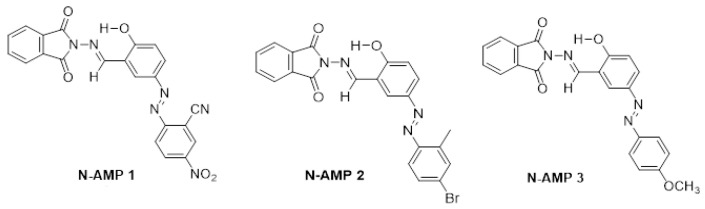
Structure formula of the studied compounds.

As a result of the above information, the aim of this report is to present the achieved results of the studies on the physicochemical properties of three N-phthalimide azo-azomethine dyes (NAA; L1, L2, L3) (Figure 1) [23] and to evaluate the possibility of formation of complex compounds with metal ions such as copper, nickel, lead and cobalt and to outline the prospects and possibilities of the studied organic ligands for their application as indicators and molecular sensors in the analysis of real systems.

## 2. Results and Discussion

### 2.1. Determination of Protonation Constants of Investigated Compounds

**Figure 2:**
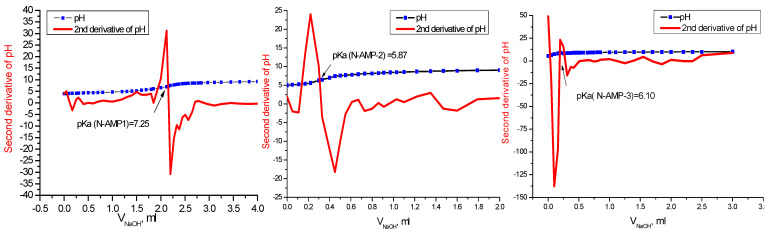
Plot of function (second derivatives of pH) = f(V_NaOH_, mL) at potentiometric titrations of 16.60 µmol L1, 15.06 µmol L2 and 19.93 µmol L3.

The quantitative evaluation of the possibility of intramolecular proton exchange of the compounds is the equilibrium proton transfer constant pKa, which is one of the physicochemical properties that determine the molecules’ basic sensor activity as well pharmacological and pharmacodynamic parameters. Given the great importance of acid–base constants as a physicochemical parameter, showing the affinity of a molecule for proton exchange, the present interest in the application of methods for the study of acid–base processes, the determination of the ratio of concentrations of equilibrium forms of a compound, and the calculation of the corresponding pKa values increase [32,33]. To determine the ratio of azo-azomethine derivatives to complexation with transition metal ions, both the behavior in aqueous solutions and their acid–base constants and complex stoichiometric coefficients were determined. As can be seen from the structure of the investigated compounds in an aqueous solution, the proton donor groups will be the hydroxyl group of the salicylic moiety and/or azomethine component. We used the potentiometric and spectrophotometric methods to determine the pKa values. The titration curve and the second derivative of pH data vs. volume of the titrant are given in Figure 2. The value of pKa was determined using the Henderson–Hasselbalch equation [34], potentiometrically by studying the functions: pH = f(V_(NaOH)_) (Figure 2) and $\mathrm{pH}=f\left(\mathrm{lg}\frac{C_{\mathrm{acid}}^{0}\times {\;V}_{\mathrm{acid}}^{0}}{C_{\mathrm{base}}^{0}\times {\;V}_{\mathrm{base}}^{0}}-1\right)\;$(Figure S1). It binds pH and pKa to the equilibrium concentrations of dissociated acid (A^–^ deprotonated form of L) and undissociated acid (HA, protonated form of L), respectively. The values of the acidity constants were calculated from the inflection points of the titration curves and from the intercept of the function $\mathrm{pH}=f\left(\mathrm{lg}\frac{C_{\mathrm{acid}}^{0}\times V_{\mathrm{acid}}^{0}}{C_{\mathrm{base}}^{0}\times V_{\mathrm{base}}^{0}}-1\right)$. The obtained results are summarized in Table 1. The ability of the compounds to donate a proton in an aqueous medium increases in the following sequence: N-AMP-1 < N-AMP-3 < N-AMP-2. The calculated pK values of the compounds show that they are not strong protolytes (acids), but the presence of electron-donating groups (for example, -OH) increases the basicity of the nitrogen atoms, which are able to bind hydrogen protons in accordance with the donor–acceptor principle.

**Figure 3:**
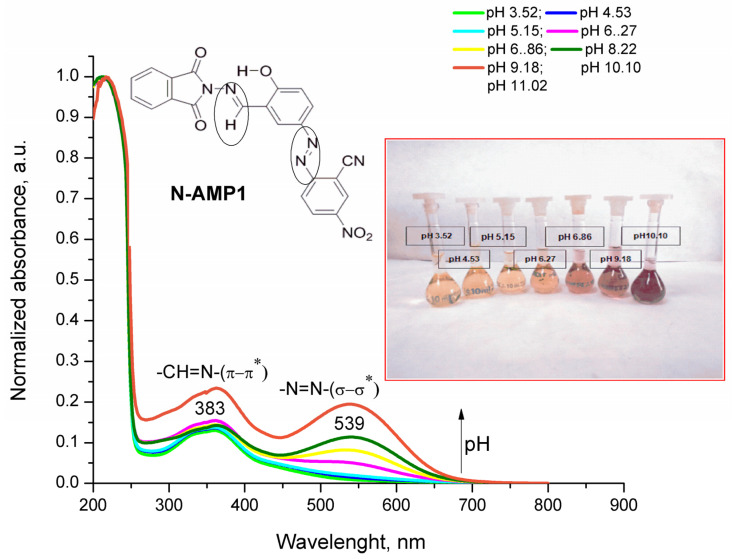
UV–Vis normalized spectra of N-AMP1 (L1) at equal concentration of analyte in each photometric solution (C = 6.81 × 10^−5^mol L^−1^) and different pH (buffer Robinson, pH 2–11).

**Figure 4:**
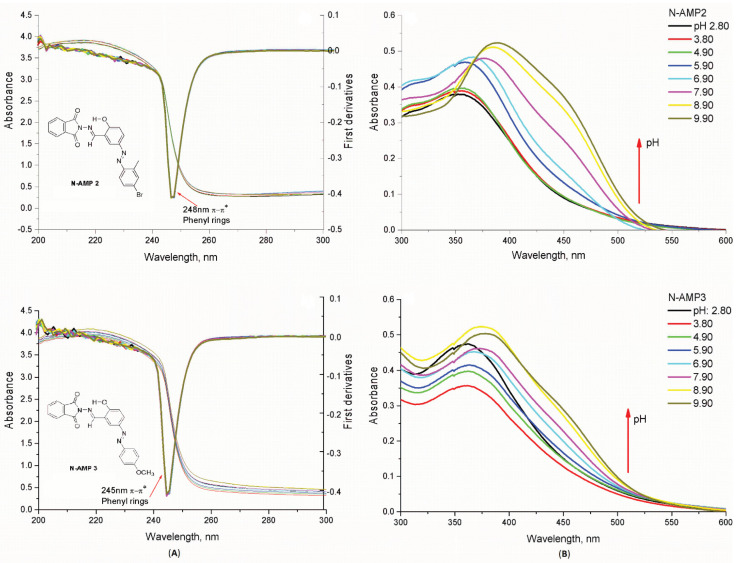
UV–Vis spectra of N-AMP2 (L2, C = 6.03 × 10^−5^ mol L^−1^) and N-AMP3 (L3, C = 7.97 × 10^−5^ mol L^−1^) at different pH (buffer Robinson, pH 2–11) and scaling as follows: (**A**) UV–Vis (zero) and differentiated spectra from 200 to 300 nm and (**B**) UV–Vis spectra from 300 to 600 nm.

The values of the pK_a_ constants were confirmed spectrophotometrically by Kumar’s method using UV–Vis data for the absorbance of the analytes in different pH (Figure 3 and Figure 4) and the follow equation:

$$K_{a}=\frac{\left(C_{H}^{i}-C_{H}^{k}\right)\times \left(A^{i}\times C_{H}^{i}-A^{n}\times C_{H}^{n}\right)-\left(C_{H}^{i}-C_{H}^{n}\right)\times \left(A^{i}\times C_{H}^{i}-A^{k}\times C_{H}^{k}\right)}{\left(A^{i}-A^{k}\right)\times \left(C_{H}^{i}-C_{H}^{n}\right)-\left(A^{i}-A^{n}\right)\times \left(C_{H}^{i}-C_{H}^{k}\right)}$$

where $C_{H}^{i},{\;C}_{H}^{k},{\;A}^{i},{\;A}^{n},{\;C}_{H}^{n},{\;A}^{k}\;$ are the concentration of hydrogen ions (C_H_, mol L^−1^) and absorbance (A) for three points: i—at low pH; k—pH at the middle and n—at high value of pH, respectively. A detailed study of the absorption of the tested dyes in aqueous solution at different pHs is given in the next section of the manuscript (Section 2.3.1). The calculated values of the constants by the two methods coincide within the confidence interval (Table 1), confirming their determination accuracy. The values of pKa constants of the azo derivatives are close to those given in the literature for compounds with similar structures [36,37,38,39,40,41].

### 2.2. Testing for Sensors Activity in Acid-Base Titration

As can be seen, with increasing pH, the intensities of the characteristic bands in the visible region also increase, and while for L2 and L3 a slight bathochromic shift is observed, for L1 a new absorption maximum appears at 539 nm (Figure 3 and Figure 4). As shown, the test compounds change their color/intensity in a proton medium with a different pH. The compounds themselves have an azo bond (-N=N-) in their compositions, which is responsible [42] for the color transition from yellow in an acidic medium to mahogany in an alkaline solution for L1 (Figure 3). It is known that indicators that change color in a slightly alkaline medium (such as phenolphthalein) are suitable for titration of weak acids, and indicators converting from one color to another in a slightly acidic medium (methyl orange), are preferred for titration on weak foundations. However, if a weak acid is titrated with methyl orange or a weak base with phenolphthalein, the results of the analysis will be greatly underestimated and an error will appear. On the other hand, with the traditionally used indicators (methyl orange, phenolphthalein) in acidic basic practice, it is difficult to capture with the naked eye the equivalent point in the titration of strong protoliths. As such, the endpoint of the titration is taken at the first color change of the solution. This is mainly because the equivalent point (at pH ≈ 7) falls outside the conversion interval of the indicator determined by the equation: pH = pKa ± 1. Thus, compounds with pK values close to pH at complete neutralization and with a visibly strong color change at different pH of the solution would be preferred in a titration of strong protoliths. From the test compounds, the azo-azomethine derivative meeting the above conditions is L1 (Figure 3 and Figure 4), with the sharpest color change from yellow to mahogany observed at pH = pK = 7.08 (Table 1). This provoked our interest to test L1 for indicator activity in acid-base titration. The obtained results are summarized in Table S1. The results show that the studied azo-azomethine derivative (L1) exhibits sensory activity to determine the equivalent point in the analysis of strong protoliths with excellent accuracy and can be successfully applied as an indicator in acid–base titration.

### 2.3. Study of Complex Formation process

#### 2.3.1. Spectral Characterization of the Complexes

**Scheme 1:**
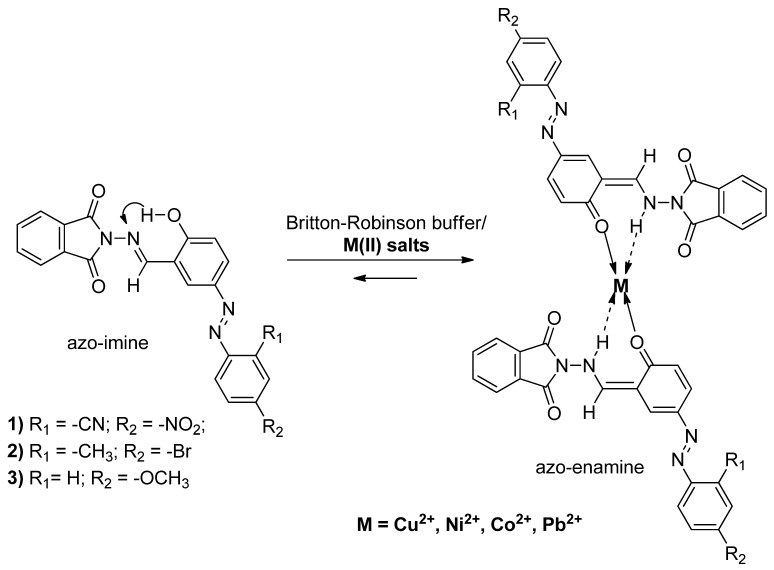
Coordination of the metal ions (Cu(II), Ni(II), Co(II), Pb(II)) in complexation with N-phthalimide azo-azomethine derivative.

**Figure 5:**
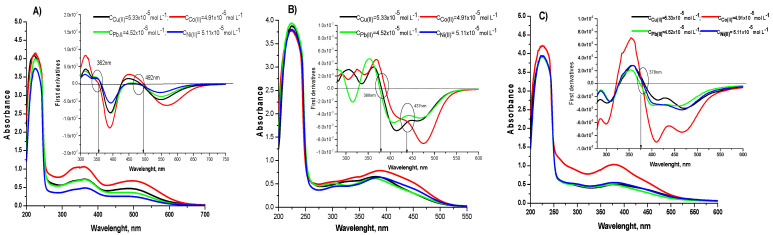
UV–Vis (zero) and first derivatives (inserted graphics) spectrum of complex equilibrium studies of the metals (Cu(II), Co(II), Pb(II), Ni(II)) with azo-azomethine derivatives: (**A**) N-AMP 1 (C = 4.88 × 10^−5^mol L^−1^); (**B**) N-AMP2 (C = 4.88 × 10^−5^mol L^−1^) and (**C**) N-AMP3 (C = 5.21 × 10^−5^ mol L^−1^) at approximately equal concentrations of the metals and ligand.

The tautomeric imine ⇆ enamine equilibrium is ensured by proton transfer of the *ortho* -OH group to the nitrogen non-bonding electron pair -CH=N- group via a six-membered intramolecular hydrogen bond and depends on the solvent polarity related to the dipole–dipole intermolecular interactions between dye and solvents [23,43,44,45]. In polar solvents, enamine forms predominate compared to the imine one. Britton–Robinson buffer (pH 9.52) is a polar medium, where the studied dyes were used as ligands for complex formation with different M(II) (Cu(II), Ni(II), Co(II), Pb(II). The structure represents two ligands coordinated to the central M^2+^ ion by enamine forms of the dyes, where an electron withdrawing (EW) keto group interacts with metal ions and an electron donating (ED) enamine group causes donor–acceptor hydrogen bonding in metals (Scheme 1). The absorption spectra of the dyes in different proton-saturated media are given in Figure 3 and Figure 4. The figures show the characteristic bands of major chromophore groups in the compounds. The band around 250 nm is due to phenyl rings and the spectra were differentiated at these bands because they do not change during the metal ion addition (Figure 4A). Absorption spectra of the dyes and their complexes show that all dyes in the presence of transition metal ions show a hypochromic effect with insignificant bathochromic (L1) and weak hypsochromic effects of main π → π* (S_0_ → S_2_) absorption bands in the long wavelength UV range, which characterized azo and azomethine chromophore groups (Figure 5 and Figures S2–S4). L1 shows strong absorption band in the VIS range (λ_max_ = 539 nm) for n → π* (S_0_ → S_1_), which indicates the enamine form (Figure 3). Upon addition of ions, the band significantly decreases due to transfer of π-electrons to the metal ions (Table 2 and Figures S2–S4). This indicates that they can be used as sensors for metal ion detection. The fluorescence properties of the dyes allow them to be exploited by ESIPT (excited state intramolecular proton transfer) as sensitizers for ion detection (Figure S5). Therefore, the fluorescence spectra of the N-AMP ligands as well their metal complexes with Cu (II), Ni (II), Co (II), Pb (II) with molar ratios ML and ML2 were recorded and are presented in Figure S5. By the addition of Cu(II) to L1 solution, strong quenching of the fluorescence emission was observed compared to the ligand solution, while for L2 and L3 dyes quenching was minimal due to the ED substituents in phenylazo moiety and d-electrons of copper, which provide photoinduced electron transfer to the enamine parts. In addition, there is a quantitative relationship between the concentration of the metal ion and the quenching of the fluorescence as at higher concentrations of metal in the complex compound the intensity of the analytical signal decreases, a phenomenon most pronounced in copper ions in NAP1 solution in a ratio of 1:2 (Figure S5). Turning off the emissions in the presence of ions is explained by blocking the ESIPT process. The spectra of L1 with Ni(II), Co(II), Pb(II) indicate similar behavior to Cu(II); however, for N-AMP2, red shifted emissions are observed, especially for Ni(II), Co(II), Pb(II). Decreasing intensities and blue shifted emissions are the main spectral features of the L3 with Ni(II), Co(II), Pb(II), compared to the Cu(II) one. Table 2 summarizes the emission and related Stokes shifts of the dyes with studied metal ions. In order to examine the applicability of the ligands as transition metals ion sensors, from the spectral data the following conclusions can be made: (i) specific fluorescence bands at λ_em_ = 428 nm of L1 and λ_em_ = 496 nm L3 are turned off with blue shifted emissions; (ii) L2 has two emissions at λ_em_ = 397 and 465 nm, where the fluorescence intensities slightly decrease compared to the others and red shifted emissions are observed.

#### 2.3.2. Determination of Complex Stoichiometry and Stability

**Figure 6:**
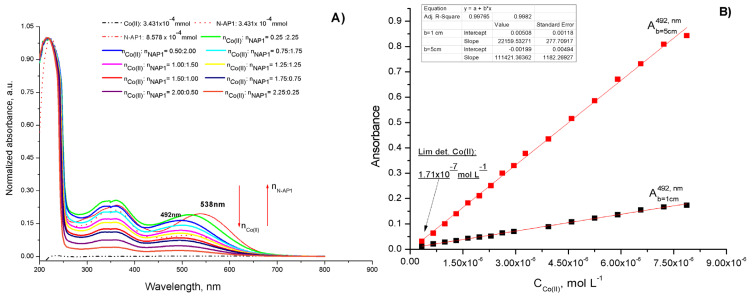
(**A**) Absorption spectra of solutions containing Co (II) and ligand: N-AMP1(L1) with different molar fractions of the two binding agents; (**B**) study of the dependence of A = f(C_Co (II),_ mol L^−1^) on solutions containing twice the amount of excess ligand.

**Figure 7:**
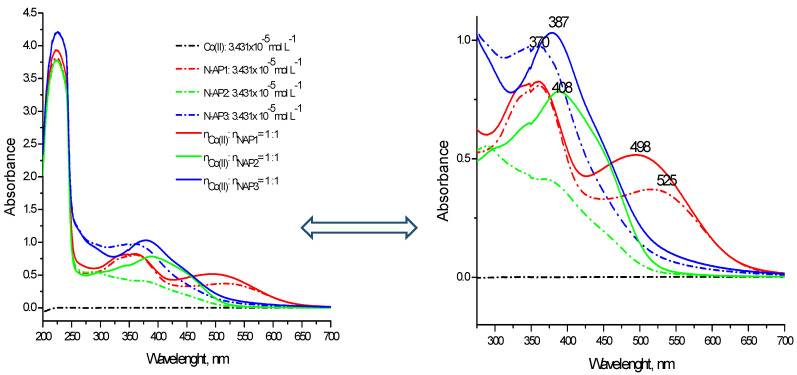
Absorption spectra of Co (II) -N-AMP(L) complexes in a ratio of 1:1 and of unbound azo compounds.

**Figure 8:**
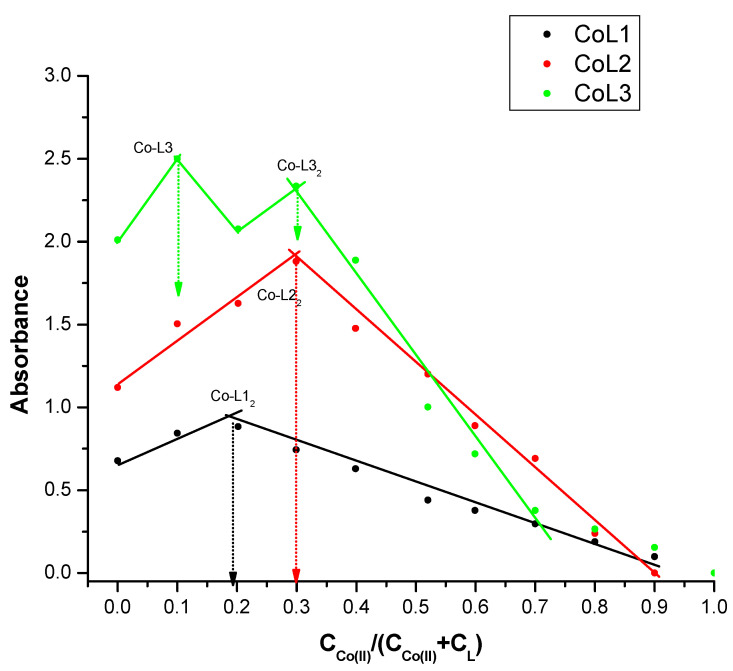
Graphical representation of the dependence A = f(C_Co(II)_/(C_Co(II)_ + C_L_) (Job method) for solutions containing different content of metal ions (Co(II)) and ligand (L1, L2 and L3).

The ratio between the metal and azo reagent is a key factor for the stability of the complex, which is important for the development of the metal determination methods, minimizing the metal ions quantities for determination. The stoichiometry of the complexes was studied using the method of continuous variations (Job’s method). Experimentally, the absorptions at a given wavelength of a series of solutions containing varying molar fractions of metal ion and ligand were measured. If the complexation equilibrium is far to the right, in the direction of formation of the complex, then the absorption will be greatest when the (L) in the solution is exactly n times greater than (М). Therefore, we can determine n (stoichiometry coefficient of the complex) and thus the composition of metal-N-AMP systems by knowing the ratio of L to M in the solution that contains a maximum absorbance of MLn (Figure 6). The M(II)-N-AMP absorbances were plotted against molar fractions of N-AMPs and metal and the prove complex stoichiometry is as follows: CuL1, CuL1_2_ (Figure S6), CuL3_2_ (Figure S7); NiL1, NiL1_2_, NiL2_2_ (Figure S7), NiL3_2_; PbL1_2_, PbL2_2_, PbL3. In Figure 6 is shown UV–Vis spectra in the case of the azo-azomethine-Co(II) chelate, which is obtained by plotting the absorbance at 538 nm (Figure 6, spectra of molar fractions). Evidence that the absorption lines are of the obtained complexes is presented in Figure 7 where it is seen that even at a molar ratio of metal ligand of 1:1, only absorbent species at 498, 408 and 387 nm are the cobalt ion–ligand complexes. The rough fit of the two straight lines intersecting at 0.36 indicates that a 1:2 complex (CoL_2_) is formed for L2 and L3 (Figure 8). As can be seen, the obtained complexes are in the ratio 1:1 or 1:2, i.e., ligand concentration is commensurate to or twice higher than the metal ion, which is sufficient for the complexing reaction to proceed. To obtain reliable results, each point of the curves was obtained as the mean of the absorbances of at least two solutions at the same concentrations. The results also showed that the absorption of the compounds increased linearly with the increase in the concentration of the metal–ligand (ML_2_) complex compound (Figure 6B). It was observed that the linearity did not change; there is no deviation from Beer’s law at low concentrations of metal ion (Figure 6B). The experimental limit of determination was determined to be at 1.71 × 10^−7^mol L^−1^ Co(II) in the solution as the sensitivity of the determinations significantly increased when measuring the absorbances of the solutions in a cuvette with a larger internal width (Figure 6B, the red line).

The stability constants of the complexes formed between ligands and metal ions, causing the strongest increase in the signal, were also calculated using data from potentiometric titration of the strong acid, the free form of the ligand in the presence of strong acid, and ligands with the presence of metal ions [46] (Figure S9). As can be seen from Table S1, the most stable complex L1 forms with Co(II) (logβ = 5.93), and L3 with Cu(II) (logβ = 6.15), respectively. From the titration curves, it can be observed that the significant deviation between acid–ligand (L1) and Co(II)–L1 curves started from pH = 4.0, indicating the commencement of complex formation.

### 2.4. Electrochemical Characterization of the Compounds and Their Metal Complex Formation

**Figure 9:**
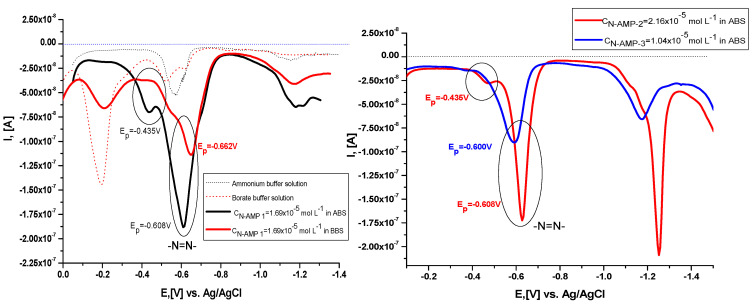
Differential pulse voltamperograms (cathodic) of investigated azo-azomethine derivatives in ammonium (pH 10.35, 0.1 mol L^−1^) and borate (pH 9.18, 0.1 mol L^−1^) buffer solutions at HMDE working electrode and Ag/AgCl, KCl (3 mol L^−1^) as reference electrode.

**Figure 10:**
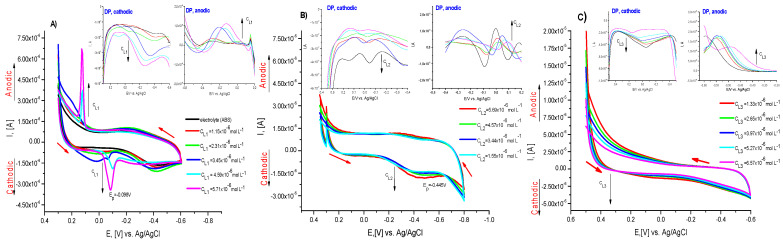
Ciclyc voltamperograms and differential pulse voltamperograms (inserted graphics) of investigated azo-azomethine derivatives (**A**) N-AMP1; (**B**) N-AMP2 and (**C**) N-AMP3 in ammonium (pH 10.35, 0.1 mol L^−1^) buffer solutions at Pt working electrode and Ag/AgCl, KCl (3 mol L^−1^) as reference electrode; scan rate: 1.00 V s^−1^.

**Figure 11:**
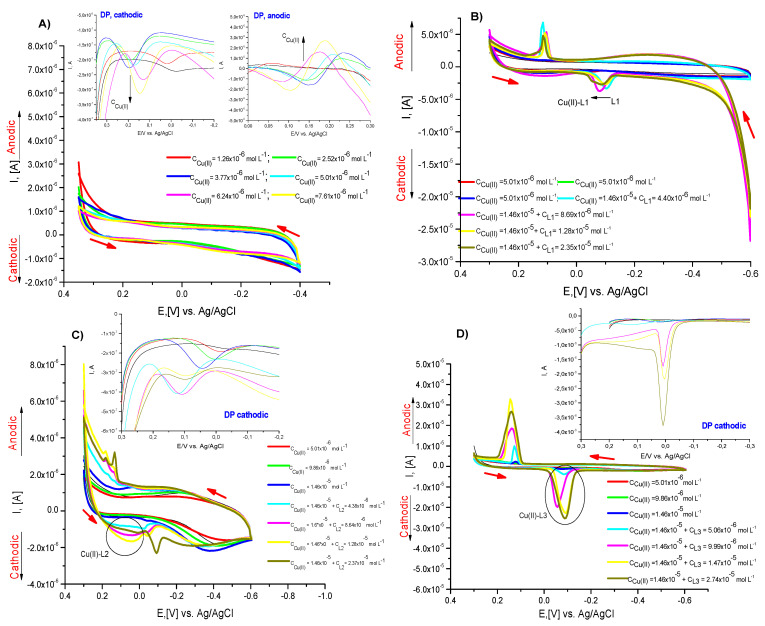
Ciclyc voltamperograms and differential pulse voltamperograms (inserted graphics) of free copper (II) ions (**A**) and Cu(II)-L complexes (**B**–**D**) in ammonium (pH 10.35, 0.1 mol L^−1^) buffer solutions at Pt working electrode and Ag/AgCl, KCl (3 mol L^−1^) as reference electrode; scan rate: 1.00 V s^−1^.

**Figure 12:**
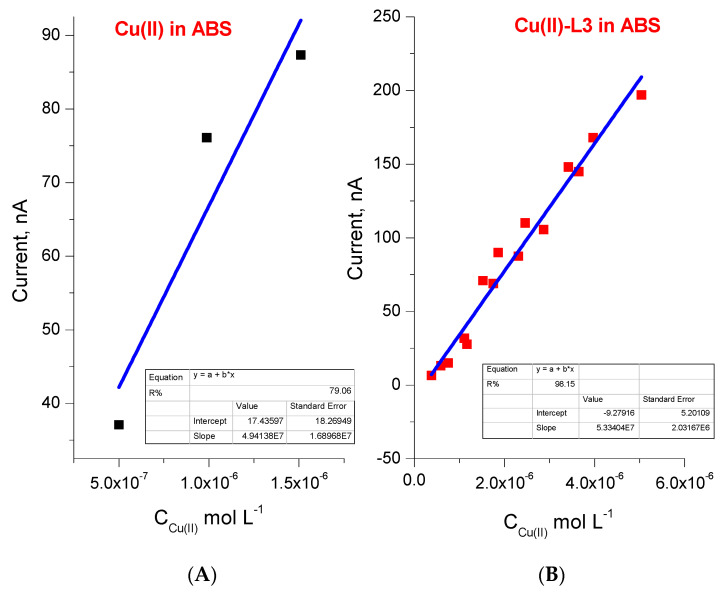
Plot of Ipc, nA vs. C_Cu(II)_, mol L^−1^ of (**A**) free Cu (II) in ammonium buffer solution (ABS) and (**B**) Cu (II)-L3 with tenfold excess of azo-azomethine derivative.

The spectral behavior of the azo-azomethine derivatives shows promising results for application, for example, as a chemosensor for metal ions in the environment. In addition to spectra, the interaction of azo-azomethine dyes with metal ions has been also proven electrochemically. A method to determine copper ions in surface waters based on sensory activity exhibited by the studied ligands has been developed. Taking into account the influence of the environment and the possibility of side reactions and the matrix influencing the sample, satisfactory results were obtained. The electrochemical behavior of the azo derivatives proved that the test compounds gave an analytical signal in an alkaline medium to both the mercury and the platinum electrodes. The alkaline environment also proved to be a favorable environment for the complexation of copper ions. It has previously been shown that electrochemical detection of copper ions in surface samples in an alkaline medium (pH 10.56, LiOH/LiOH) is possible without the medium affecting the stability of the complex [47]. The compounds gave an electrochemical response in a polar solvent, showing a signal different from that of the pure electrolyte medium at both electrodes. The analyses were performed on both electrodes, as mercury electrodes are classic detectors of metals in different media, but due to their toxicity, their replacement with “eco-friendly” electrodes is increasingly preferred. There are a limited number of developed electrochemical methods in the literature for the determination of metal ions in aqueous samples using a platinum working electrode and the participation of an azo-azomethine sensor for signal detection or amplification. Thus, the recorded cathodic signals of the initial compounds of the Hg and Pt electrode in ammonia and borate buffer solutions show well-formed cathode peaks localized at potential E_pc_ ≈ −0.600 V, which can be attributed to the reduction of the -N=N- group to -NH-NH- (Figure 9). A second peak at the more negative potentials ≈−1.20 V was observed in the proton solution, which can be bound to the nitro group of N-AMP1 and/or to the azomethine bond in all compounds [31,36]. In the cyclic mode of the Pt- electrode, the cathodic reduction (at E_p,c_ ≈ −0.100 V) and the reverse anodic oxidation (at E_p,a_ ≈ +0.100V) of the azo bond of L1 were also proved (Figure 10). The behaviors of the other two ligands (L2 and L3) were quite different (Figure 10B,C). For L2, a cathode peak was recorded at −0.445 V; L3 did not give an electrochemical response to the studied concentration range and voltammetric mode. Low-intensity current signals (anodic and cathodic) were observed in the differential pulse mode, with no proportional increase in current intensity with increasing analyte concentration (Figure 10B,C, inserted graphics; Figure S10). When studying the influence of the different metal cations on the electrochemical parameters of azo dyes using a mercury electrode, it was found that the current peak intensity increases in the presence of analytes and/or shifts to more negative potentials due to complexation between the metal ion and ligand (Figure S11 and Table S3). The strongest displacement, proving the formation of a stable complex at the platinum electrode, was observed in the Co(II)-L1 complex, and the most effective detection response associated with a significant increase in the current peak was shown by L3 upon its binding to copper ions. This is evidence for a complex formation process and an opportunity to study sensory activity of the new azo ligands. Generally, the application of small molecular detectors for metal ion detection in surface water samples presents a unique set of challenges, which requires detailed studies of sensor performance in the environmental milieu and method/device design. Figure 11A shows the voltammograms (cyclic and differential pulse) of direct detection of Pt electrode copper ions in an ammonia medium. It can be seen that in the concentration range from 1 × 10^−6^ to 8 × 10^−6^ mol L^−1^ in the cyclic mode, there was no significant increase in the signal with the increase in the analyte concentration. There is also no proportionality of the current intensity with an increasing concentration in the DP mode (Figure 11A, inserted graphic). When the tested ligands were added, a change in the analytical signals was seen—a proportional increase in the current intensity, as the correlation coefficient of the dependence I_C_ vs. C_Cu(II)_ is the highest in value when using NAP3 (L3) as a binding compound (Figure 12).

### 2.5. Sensor Activity Test toward Determination of Cu(II) in Environmental Samples

Heavy metals contained in the surface waters of river systems are subject to continuous monitoring, as them being in quantities above the specified national legislation poses a serious environmental and health problem due to their function of accumulating toxins in the human body, falling directly from water sources used for irrigation and drinking needs. Extremely fast and cheap control of the metal concentration in water is provided by the methods of voltammetry with the use of chemical sensors, as the main analytical characteristics (accuracy, precision, limit of detection and determination) of the measurements are comparable and in some cases better than those of widely used spectroscopic methods. As a result of previous developments, only a limited number of all proposed molecular sensors for metal ions have a practical implementation, and the main limitations in the analysis of real systems stem from the requirements for high chemical stability, good selectivity and fast response. Electrochemical analysis of various substrates based on the interaction between the analyte and the sensor molecule is one of the most sensitive methods. The selectivity of the assay is ensured by the ligand used, which selectively interacts with a given substrate. As a result of this interaction, changes occur in the electrochemical characteristics of the electron exchange fragment. Precisely due to the fact that this interaction takes place at the molecular level, extremely low concentrations of analytes can be detected by this method, which makes it highly effective. Thus, the sensory activity of the test compounds (L3) was tested in the determination of copper ions in real surface water samples using the complexing properties of the metal ion and electrochemical detection. The potential of the studied compounds as ligands for the detection of copper ions was studied in an ammonia medium, on a platinum electrode by monitoring the changes in the voltammograms in the presence and absence of copper ions. The choice of ABS as a medium for measurements is due to the fact that both the initial ligands and metal ions and the complexes formed between them exhibit good electrochemical characteristics. After the addition of an amount commensurate to the metal to a tenfold excess, a shift of the signal of the azo component to the more negative potential windows is observed. As can be seen from Figure 11, the intensity of the new peak at −0.237 V is lower than that of free copper and the intensity of the peak at −0.653 V obtained after complexation. The behavior of the analytic signals of the complex at different concentrations of copper and a minimum of tenfold excess of ligand is also presented (Figure 11). Figure 12 presents the relationship between the current intensity and the concentration of Cu(II), as well as between the peak of the current and the concentration of the formed complex. As can be seen from the graphic, a better linear relationship (R = 0.981) was found between the concentration of the complex and the current at a metal ion concentration in the region from 3.7 × 10^−7^ to 5.0 × 10^−6^ mol L^−1^ and a twofold excess of ligand. The quantification (LOQ) and detection (LOD) limits were determined by the equation: Cmin = 10 × Sb/m, and 3 × Sb/m (where Sb is the standard deviation at *n* = 6 replicate measurements of the blank sample and m is the slope of the calibration curve) [48]. For the analyte, the LOD value was from 5.63 × 10^−8^ mol L^−1^ (3.58 µg L^−1^) and LOQ: 1.88 × 10^−7^ mol L^−1^ (11.9 µg L^−1^) [47], respectively. The k-coefficient of the analytical function I = k.C described by the slopes of the calibration curve are as follows: 4.91 × 10^7^ nA.L mol^−1^ (for free copper) and 5.33 × 10^7^ nA.L mol^−1^ (for Cu-L3). As can be seen, the sensitivity of the determinations is improved in the determination of copper ions using the molecular sensor and electrochemical detection at Pt electrode (Figure 12).

### 2.6. Analytical Performances of the Method 

When testing the sensory activity of L3 in real surface water samples, two samples from nodal sections of the Topolnitsa River were analyzed by at least three replicates of each analyzed sample. The results for copper content obtained both from the electrochemical detection as a result of the sensory activity of L3 and from the comparative method are as follows: sample S1: (3.91 ± 0.02) × 10^−4^ mol L^−1^ and S2: (2.45 ± 0.04) × 10^−4^ mol L^−1^, respectively. The results obtained are accurate and there is no systematic error in the definitions, as the results obtained by electrochemical detection are in the range of the confidence interval of the result obtained by the comparative ICP-OES method. The standard deviation expressing the accuracy of the method is 1.5%. The actual surface water sample has complex matrix contents and often a palette of metal contents is found. To evaluate the effect on the analytical signal of the copper–ligand complex in the presence of common ions in the water, voltammograms were recorded in the presence of various possible interferences. It was found that concentration from the following components 100 mg L^−1^ K^+^ and Na^+^; 500 mg L^−1^, Cl^−^ and NO_3_^−^, SO_4_^2−,^ PO_4_^3−^; 200 mg L^−1^ Ca^2+^ and Mg^2+^; 50.0 mg L^−1^ AL3^+^, Zn^2+^, Fe^2+^, Fe^3+^ did not affect the determination of minor and trace copper. The interference studies were carried out in model solutions by adding a known amount (0.150 mL) of coexisting species to Cu-L3 solutions, containing 2.0 × 10^−6^ mol/L copper ions. The first nine of the listed potential indifferent ions do not give a direct signal under the indicated conditions (electrolyte medium and working electrode) and do not interact with the ligand, due to which the current peak of the analyte was not affected. The indicated concentrations of Zn(II) and both forms of Fe had no interfering effect due to the fact that zinc ions in the ammonia medium were registered at ≈ −1 V and no signal was observed for the reduction of Fe(III) to Fe(II) as a complex between Fe(III) and L3 probably formed, in which the signal shifts strongly in the negative direction. The determination of method selectivity was also carried out following the procedure given in [47]. The recovery (R, %) after spike of the sample was determined in order to evaluate the influence of all matrix components of the samples and the recovery results are presented in Table 3. The obtained recovery values for both samples prove good accuracy and selectivity of the method of electrochemical detection of Cu (II) ions using the molecular sensor L3 [47]. 

### 2.7. Reagents and Solutions

The synthesis pathway of used N-phthalimide azo-azomethine dyes (N-AMPs: N-AMP1 (L1), N-AMP2 (L2), and N-AMP3(L3), Figure 1) is given in [23]. Britton–Robinson (0.04 mol L^−1^; pH 2–11), Borate (BBS; 0.1 mol L^−1^; pH 9.18 ± 0.01) and Ammonium (ABS; 0.1 mol L^−1^; pH 10.35 ± 0.01) buffer solutions, Cu(NO_3_)_2_.3H_2_O, Ni(NO_3_)_2_.6H_2_O, Pb(NO_3_)_2_ and Co(NO_3_)_2_.6H_2_O (Sigma Aldrich, analytical grade with high purity > 99%) were used without further purification. The all buffer solutions were adjusted to 0.1 mol L^−1^ ionic strength. The various stock solutions of metal ions were prepared with double-distilled water with concentrations as follows: Cu(II) 0.09819 mol L^−1^; Pb(II): 0.1208 mol L^−1^, Ni(II): 0.1012 mol L^−1^ and Co(II)—0.1009 mol L^−1^, respectively. The values of the ligand stock solution concentrations were: 3.317 × 10^−3^ mol L^−1^ L1; 3.013 × 10^−3^ mol L^−1^ L2; 3.986 × 10^−3^ mol L^−1^ L3, prepared by dissolution of the *N*-phthalimide azo-azomethine dyes in dimethylformamide (DMF). The working solutions for electrochemical and spectroscopic analyses of all reagents were prepared by appropriate dilution of the stock solutions with water. 

### 2.8. Spectral Measurements

The UV–Vis spectra of the dyes and their metal complexes (Cu(II), Ni(II), Co(II), Pb(II)) were collected on the Cary 5E- UV–VIS-NIR spectrophotometer at room temperature in Britton–Robinson buffer (pH from 2 to 12). The fluorescence spectra were recorded via a FluoroLog 3–22 spectrofluorometer in the range 200–800 nm with resolution of 0.5 nm and double-grating monochromators by excitation wavelength near to the absorption maxima of dyes. 

### 2.9. Electrochemistry 

#### 2.9.1. Instrumentation

The analytical signals (voltammograms) were recorded on a computer-controlled voltamperometer Metrohm 797 VA trace analyzer with a 797 VA stand connected to a three-electrode cell. The electrode system consists of a single-body working electrode (hanging mercury drop electrode (HMDE) or Pt solid electrode) with a Ag/AgCl reference electrode in 3 mol L^−1^ aqueous solutions of KCl and an auxiliary carbon electrode. During the registration of the current–potential curves, the solution in the cell was not stirred. All the voltammetric experiments were conducted in a high-purity nitrogen atmosphere at room temperature (25 ± 1 °C). 

#### 2.9.2. Voltammetric Procedures

A three-step voltammetric procedure was applied as follows: (1) 7.0 mL of the supporting electrolyte (0.1mol L^−1^ ammonium/borate buffer solution) was placed in the electrochemical cell; (2) the aliquot of the standard analyte solutions of the azo-azomethine derivatives and metal ions (Cu(II), Pb(II), Ni(II), Co(II)) and/or interfering ion were subsequently added; (3) and their analytical signal/current–potential curves were registered. Before each signal was recorded, the solution in the electrochemical cell was degassed with nitrogen for 200 s. The presented results are reported as the mean value of three independent measurements.

#### 2.9.3. Potentiometric Measurements

A digital pH meter (Janwey) combined with a glass electrode, a magnetic stirrer and a semi-micro-burette with divisions of 0.01 mL were used for potentiometric measurements of pH of the buffer solutions, pK constants and determination of complex stability. For each titration, 3.00 mL from stock solutions of the N-phthalimide azo-azomethine dyes were titrated with standardized base (0.01220 mol L^−1^ NaOH). Prior to measurement, the pH apparatus was calibrated, according to standard methods, with two buffer solutions to a constant value of the measured physical quantity. Data (volume of titrant vs. pH) were processed by Origin8Pro software. 

### 2.10. Testing for Indicator Activity 

The acid–base titrations were carried out at 25 °C as follows: (1) analyte: strong acid (HCl: 20.00mL 0.1021 mol L^−1^) vs. strong base as a titrant 0.09921 mol L^−1^ NaOH; (2) Analyte: strong base (NaOH: 20.00 mL 0.09921 mol L^−1^) vs. strong acid as a titrant 0.1021 mol L^−1^ HCl. A solution (≈1%) of the compound tested for indicator activity was prepared as 0.1 g of starting substrate was dissolved in 10 mL of DMF. Five drops of indicator solution were needed to color the titrated solution. 

### 2.11. Testing for Sensor Activity toward Copper Ions in Water Samples 

Sampling and Sample Location of the Water Samples 

The studied surface water samples from the catchment area of the Topolnitsa River (Bulgaria) were collected, preserved and analyzed according to standard methods [30]. The samples were kept in a 250 mL plastic container after adding of 3 mL of nitric acid. The sampling stations and locations of investigation samples are given in Table 4. The full chemical compositions (Table 5) of the samples were determined by standard ICP-OES method (Inductively Coupled Plasma Optical Emission Spectroscopy, method: ISO 011885) using the Prodigy High Dispersion ICP-OES, Teledyne Leeman Labs, USA spectrometer with the follow measured parameters: a dual-view torch, cyclonic spray chamber, and concentric nebulizer: coolant gas—18 L/min, auxiliary gas—0.5 L/min, nebulizer gas—34 psi, RF power—1.2 kW, pump rate—1.2 mL/min, sample uptake time—30 s, integration time—40 s. High purity Ar 99.999% was used to sustain plasma and as a carrier gas [31]. For determination of the coefficient, the analytical function multielement standard solution (“Ultra scientific”, Lot: P00332) containing 24 elements in 5% HNO_3_ (Al, Bа, Bе, Bi, B, Cd, Ca, Cr, Co, Cu, Ga, Fe, Pb, Li, Mg, Mn, Ni, K, Se, Na, Sr, Te, Tl, Zn) at a concentration of 100 ± 5 mg L^−1^ of each element, and 2) arsenic standard solution (As in 5% HNO_3_) 1000 ± 3 mg L^−1^ (VHG Labs, Lot: 112-0017) was used [31]. The methods for standard addition and calibration curve were chosen to determine the concentration of the target analytes in the samples.

### 2.12. Procedure for Metal Determination 

The metal determination procedure was performed in three steps: (1) in the voltammetric cell (50.0 mL), 7.0 mL of ammonia buffer solution, a proper volume of ligand (N-AMP3, L3) and 0.100 ÷ 0.150 mL of the analyzed water samples were added; (2) after purging for 300 s with pure nitrogen, the analytical signal corresponding to the Cu(II)–L3 complex was registered (3) A mixture solution (calibration solution) from copper ions and *N*-phthalimide azo-azomethine (N-AMP3) in a ratio of 1:10 was prepared and the current-potential relations were recorded. An external standard method for the determination of the coefficient of analytical function I = f (C_Cu_, mol L^−1^), where “I” is the current in the cyclic voltamperometry, was used. The copper concentration was calculated by applying a standard additive method as aliquot volumes from calibration solution were added into working solution and the cathode and anodic peaks were read after each increase in the concentration of the analyte. The copper concentration in the sample was calculated by the formula:

$$C_{\mathrm{Cu}\left(\mathrm{II}\right)\mathrm{sample}}=\frac{a_{\left(\mathrm{intercept}\right)}}{b_{\left(\mathrm{slope}\right)}}$$

where “a” and “b” are the coefficients of the analytical function.

## 3. Conclusions

In summary, the sensor activity of three azo-azomethine derivatives toward detection of the equivalent point in the analysis of strong protoliths as well as of metal ions has been investigated in different environments and using different methods. Physicochemical characteristics such as pK, pH interval of color conversion of azo-azomethine compounds, and complex stoichiometry and stability determined by potentiometry and spectral methods are presented. Fluorometric data confirmed complexation and proved the coordination of the metal ions in the molecule of the sensors. The most likely structure represents two ligands coordinated to the central M(II) ion by enamine forms of the dyes, where electron withdrawing (EW) keto group interacts with metal ions and the electron donating (ED) enamine group forms metals donor–acceptor hydrogen bonds. The sensory activity of the studied ligands was tested in model solutions and real samples of surface water by electrochemical detection, corresponding to the changes in the solution as a result of complexation and use of mercury and platinum electrodes with different voltamperometric techniques. L3 showed the most effective sensory activity for detecting and quantifying copper ions in aqueous samples. The results for the copper content are accurate and precise, with a standard deviation of 1.57%.

## Figures and Tables

**Table 1:** Values of pKa determination of investigated compounds.

Compound	pK Determination by Potentiometry; pH = f(V_NaOH_)	pK Determination by Potentiometry: $pH=f\left(lg\frac{C_{acid}^{0}\times V_{acid}^{0}}{C_{base}^{0}\times V_{base}^{0}}-1\right)$	pK Determine by UV–Vis [35]	Mean pKa Value
NAP-1	7.25 ± 0.15 *	6.89	7.09 ± 0.08	7.08
NAP-2	5.87 ± 0.08	5.57	5.75 ± 0.04	5.73
NAP-3	6.10 ± 0.08	6.32	6.21 ± 0.06	6.21

* Confidence intervals are calculated from at least three replicates of the determinations by each method.

**Table 2:** Experimental determined Stokes shift of the dyes and their metal complexes (Cu(II), Ni(II), Co(II), Pb(II)).

Compounds	λ_abs_ (nm)	λ_em_ (nm)	Stokes Shift (cm^−1^)
N-AMP1	362	428	4260
N-AMP1 + Cu(II)	363	431	4340
N-AMP1 + Ni(II)	364	412	3200
N-AMP1 + Co(II)	362	410	3230
N-AMP1 + Pb(II)	363	411	3217
N-AMP2	398	397	3620
N-AMP2 + Cu(II)	383	397	920
N-AMP2 + Ni(II)	389	436	2770
N-AMP2 + Co(II)	387	436	2900
N-AMP2 + Pb(II)	377	423	2884
N-AMP3	385	496	5812
N-AMP3 + Cu(II)	383	496	5950
N-AMP3 + Ni(II)	381	492	5920
N-AMP3 + Co(II)	378	493	6180
N-AMP3 + Pb(II)	377	490	6111

**Table 3:** Percentage recovery of Cu(II) in spiked sample, using spiking levels of 1.021 × 10^−5^ mol L^−1^ (S1 sample: 2.776 × 10^−6^ (in 0.050 mL) and 8.329 × 10^−6^ (in 0.100 mL) mol L^−1^; S2 sample: 1.775 × 10^−6^ (in 0.050 mL) and 3.550 × 10^−6^ (in 0.100 mL) mol L^−1.^).

Sample/Volume for Analysis, mL	Concentration of *Cu(II)* (Real Sample + Amound Added), mol L^−1^	Concentration Obtained, mol L^−1^	Concentration Recovered, mol L^−1^	Recovery (R, %) *, %	Relative Error, %
S1/0.050 mL	1.299 × 10^−5^	1.312 × 10^−5^	1.034 × 10^−5^	101.3	1.3
S1/0.100 mL	1.854 × 10^−5^	1.834 × 10^−5^	1.001 × 10^−5^	98.05	−1.9
S2/0.050 mL	1.198 × 10^−5^	1.208 × 10^−5^	1.031 × 10^−5^	100.9	0.4
S2/0.100 mL	1.376 × 10^−5^	1.388 × 10^−5^	1.033 × 10^−5^	101.2	1.2

* R = (C_a+spaike_ − C_a_)/C_spike_, where C_a+spike_ is the concentration of Cu(II) after spiking, C_a_—the concentration of Cu(II) in the sample and C_spike_—concentration of analyte in spike.

**Table 4:** The sampling stations.

Sampling Stations	Location
S1: Topolnitsa River, before Pirdopska River	GPS: N42 39 27 E24 08 15
S2: Medets’ka River at HPP: (hydroelectric power station)	GPS: N42 39 05 E24 09

**Table 5:** Chemical metal composition of surface water samples determining by the ICP-OES method, mg L^−1^.

Metal Component		Al	Ba	Be	Bi	Cd	Ca	Cr	Co	Cu
Concentration, mg L−1	S1	35.69(0.04) *	<0.010	<0.005	<0.010	<0.002	122.8(0.7)	0.012(0.000)	0.423(0.002)	24.70(0.03)
	S2	20.86(0.06) *	<0.010	<0.005	<0.010	<0.002	118.9(0.8)	0.015(0.000)	0.261(0.002)	15.79(0.04)
Metal Component		Fe	Pb	Mg	Mn	Ni	K	Se	Na	Sr
Concentration, mg L−1	S1	0.583(0.003)	<0.010	111.7(0.3)	5.678(0.004)	0.126(0.002)	4.839(0.007)	<0.010	20.60 (0.06)	0.787(0.001)
	S2	0.512(0.003)	<0.010	80.21(0.04)	3.674(0.004)	0.088(0.002)	7.559(0.011)	<0.010	27.60 (0.06)	0.637(0.001)
Metal Component		Sb	As	Mo	P	Si	Tl	S	Sn	Zn
Concentration, mg L−1	S1	<0.010	<0.010	<0.010	<0.010	7.898(0.005)	<0.010	324.1(1.4)	0.075(0.003)	1.445(0.004)
	S2	<0.010	<0.010	<0.010	<0.010	5.866(0.005)	<0.010	232.5(1.5)	0.114(0.004)	0.934(0.003)

* In the brackets is given the standard deviation (SD) calculated from at least three results (*n* = 3).

## Supplementary Materials

The following are available online, Figure S1. Plots of pH vs. (lg $\frac{C_{\mathrm{acid}.\;}^{0}\times V_{\mathrm{acid}}^{0}}{C_{\mathrm{base}}^{0}\times V_{\mathrm{base}}^{0}}-1$) at titrations of 16.60 µmol L1, 15.06 µmol L2 and 19.93 µmol L3; Table S1. Mean Volume of base/acid consumed in various titrations; analytical concentrations of the titrated protoliths are as follows: CNaOH = 0.09921 mol L−1; CHCl = 0.1021 mol L−1; Figure S2. UV–Vis (zero) spectrum of complex equilibrium studies of metals (Cu(II), Co(II), Pb(II), Ni(II)) with azo-azomethine derivatives: N-AMP-1 (C = 4.88 × 10−5 mol L−1) at approximately equal concentrations of metals and ligand; Figure S3. UV–Vis (zero) spectrum of complex equilibrium studies of metals (Cu(II), Co(II), Pb(II), Ni(II)) with azo-azomethine derivatives: N-AMP-2 (C = 4.88 × 10−5 mol L−1) at approximately equal concentrations of metals and ligand; Figure S4. UV–Vis (zero) spectrum of complex equilibrium studies of metals (Cu(II), Co(II), Pb(II), Ni(II)) with azo-azomethine derivatives: N-AMP-3 (C = 5.21 × 10−5 mol L−1) at approximately equal concentrations of metals and ligand; Figure S5. Fluorescence spectra of the N-AMPs (from left to right) and their metal complexes with Cu(II), Ni(II), Co(II), Pb(II) (from top to bottom) with molar ratios of metal: ligand = 1:1 (the red lines) and 1:2 (the blue lines); the concentrations of the central ions: 3.238 × 10−5(Cu(II)), 8.52 × 10−5(Ni(II)), 8.89 × 10−5(Pb(II)) and 3.431 × 10−5(Co(II)) mol L−1 at 0.25 mL from each ion; Figure S6. UV–Vis absorption spectra of solutions containing Cu (II) and ligand: N-AMP1(L1) with different molar fractions of the two binding agents; Figure S7. UV–Vis absorption spectra of solutions containing Cu (II) and ligand: N-AMP3(L3) with different molar fractions of the two binding agents; Figure S8. UV–Vis absorption spectra of solutions containing Ni (II) and ligand: N-AMP2(L2) with different molar fractions of the two binding agents; Figure S9. Plots of pH vs. VNaOH obtained from potentiometric data at determination of the stability constants of metal–L1 complexes. The molar ratio in the solution containing metal is: metal ion: ligand = 1:2; Table S2. Values of constant formation and their logarithmic (logβ) of obtained complexes of azo-azomethine derivatives with metal ions at molar ratio metal: ligand = 1:2; Figure S10. DP anodic voltammetry at Pt working electrode in 0.1 mol L−1 acetate buffer as a supporting electrolyte and equal concentrations of the ligands: 5.71 × 10−6 mol L−1(L1), 5.69 × 10−6 mol L−1(L2), 6.57 × 10−6 mol L−1(L3); Figure S11. Differential pulse voltamperograms (cathodic) of azo-azomethine metal complex compounds in ammonium (pH 10.35, 0.1 mol L−1) buffer solutions at HMDE working electrode and Ag/AgCl, KCl (3 mol L−1) as reference electrode; Table S3. Values of potentials (Ep, (V)) and current intensities (Ip, (A)) of electroactive components (metal–ligand and unbound forms: metal and ligand) in ammonium (pH 10.35, 0.1 mol L^−1^) and borate (pH 9.18, 0.1 mol L^−1^) buffer solutions used as electrolyte medium at mercury working electrode in DPP mode.

## Sample Availability

Samples of the compounds are available from the authors.
