# Supplementary material for: Complex Activity and Sensor Potential toward Metal Ions in Environmental Water Samples of N-Phthalimide Azo-Azomethine Dyes
Source: Molecules. 2021 Sep 28;26(19):5885. doi: 10.3390/molecules26195885 (PMC8513033; doi:10.3390/molecules26195885)
Supplement: Supplementary file 1 [file molecules-26-05885-s001.zip › molecules-1379375-supplementary.pdf]

Complex activity and sensor potential toward metal ions in environmental water samples of N-phthalimide azo-azomethine dyes

Stela Georgieva[1*], Artem Bezfamilnyi[1], Anton Georgiev[2,3] and Marian Varbanov[4]

[1] Department of Analytical Chemistry, University of Chemical Technology and Metallurgy, 1756 Sofia, Bulgaria, e-mail: st.georgieva@uctm.edu

[2] Department of Organic Chemistry, University of Chemical Technology and Metallurgy, 1756 Sofia, Bulgaria, antonchem@uctm.edu

[3] Institute of Optical Materials and Technologies, Bulgarian Academy of Science, 1113 Sofia, Acad. G. Bonchev avenue, bldg. 109, Bulgaria, antonchem@uctm.edu

[4] National Institute of Geophysics, Geodesy and Geography, Department of Geography – Bulgarian Academy of Sciences (NIGGG-BAS), Sofia, Bulgaria, str."Acad. G.Bonchev", bl.3, Sofia 1113, Bulgaria,
marian.varbanov@gmail.com

* Correspondence: st.georgieva@uctm.edu; Tel.: +359 8163400; S.Georgieva

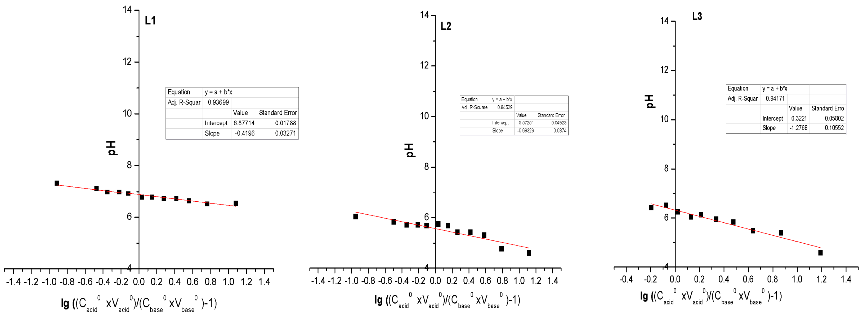

Figure S1. Plots of pH vs. $(\lg \frac{C^0_{acid.} \cdot V^0_{acid}}{C^0_{base} \cdot V^0_{base}} - 1)$ at titration of 16.60 µmol L1, 15.06 µmol L2 and 19.93 µmol L3

Table S1. Mean Volume of Base/acid consumed in various titrations; analytical concentrations of the titrated protoliths are as follow: C_{NaOH}=0.09921 mol L^{-1}; C_{HCl}=0.1021 mol L^{-1}.

Titrated protolyte	Mean experimental value	Theoretical value	Colour Change of the indicator
**NaOH**	**consumable titrant (HCl): 19.42±0.02**	**19.43**	**mahagon to yellow**
**HCl**	**consumable titrant (NaOH): 20.55±0.03**	**20.58**	**yellow to mahagon**

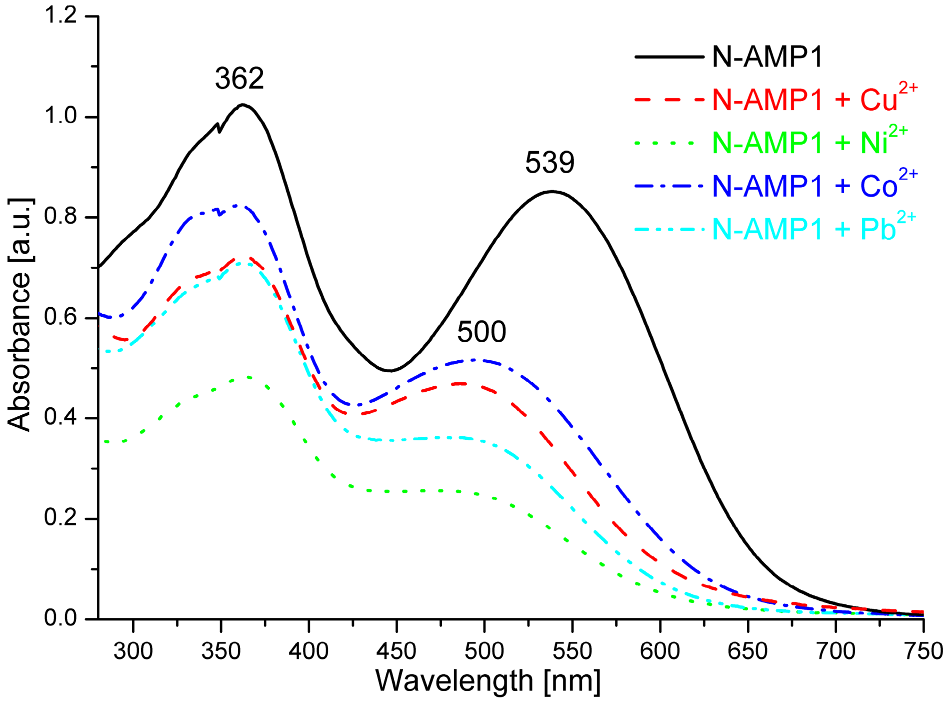

Figure S2. UV / Vis (zero) spectrum of complex equilibrium studies of metals (*Cu(II)*, *Co(II)*, *Pb(II)*, *Ni(II)*) with azo-azomethine derivatives: N-AMP -1 (C=4.88x10^{-5}mol L^{-1}) at approximately equal concentrations of metals and ligand.

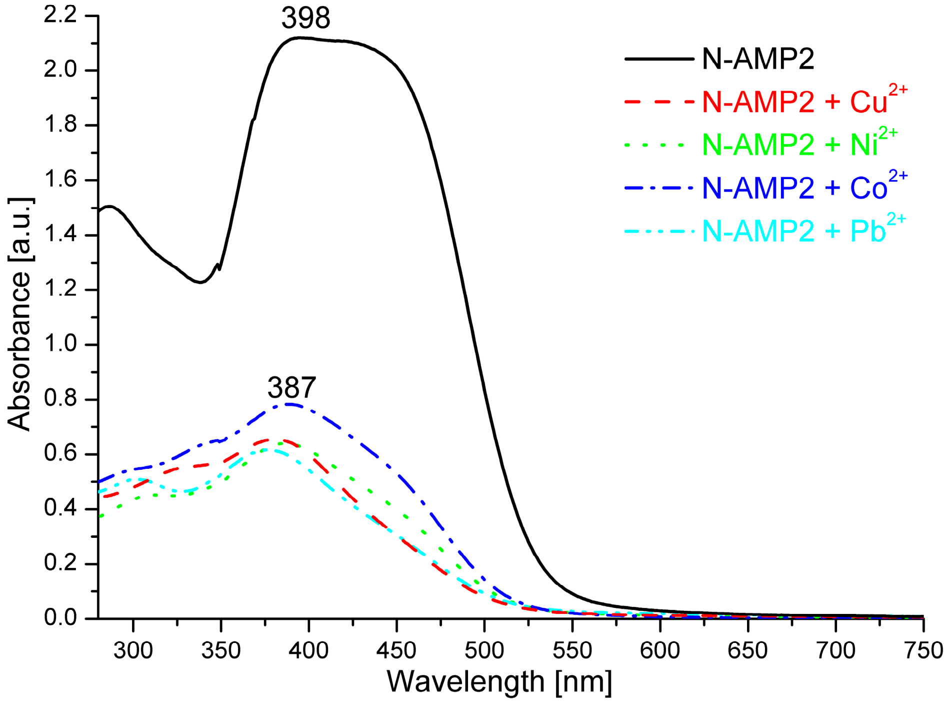

Figure S3. UV / Vis (zero) spectrum of complex equilibrium studies of metals (*Cu(II)*, *Co(II)*, *Pb(II)*, *Ni(II)*) with azo-azomethine derivatives: N-AMP -2 (C=4.88x10^{-5}mol L^{-1}) at approximately equal concentrations of metals and ligand.

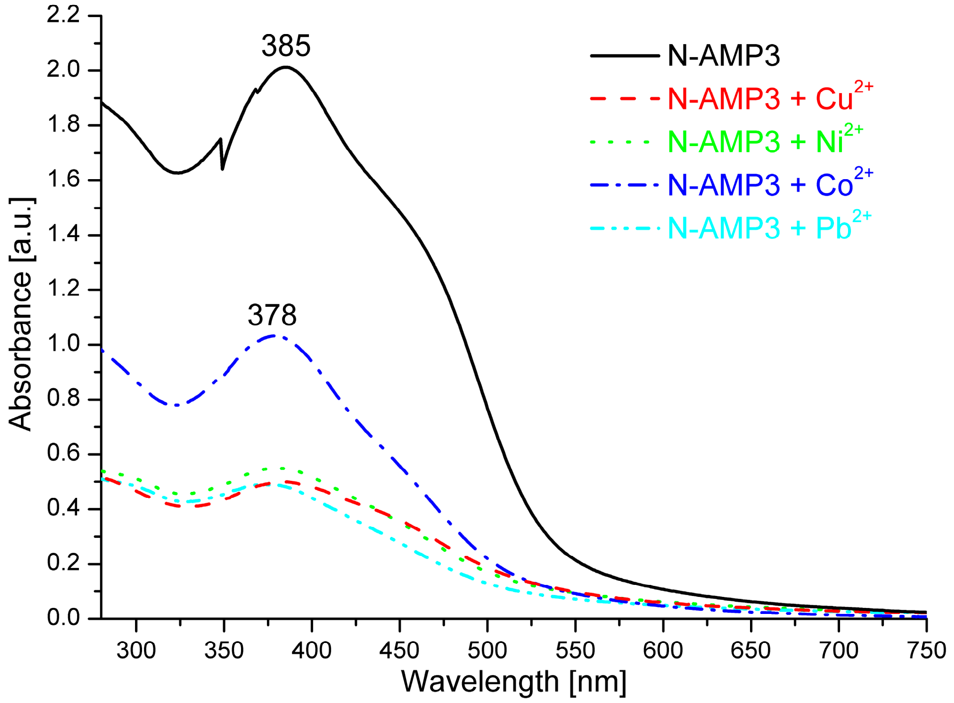

Figure S4. UV / Vis (zero) spectrum of complex equilibrium studies of metals (*Cu(II)*, *Co(II)*, *Pb(II)*, *Ni(II)*) with azo-azomethine derivatives: N-AMP -3 (C=5.21x10^{-5} mol L^{-1}) at approximately equal concentrations of metals and ligand.

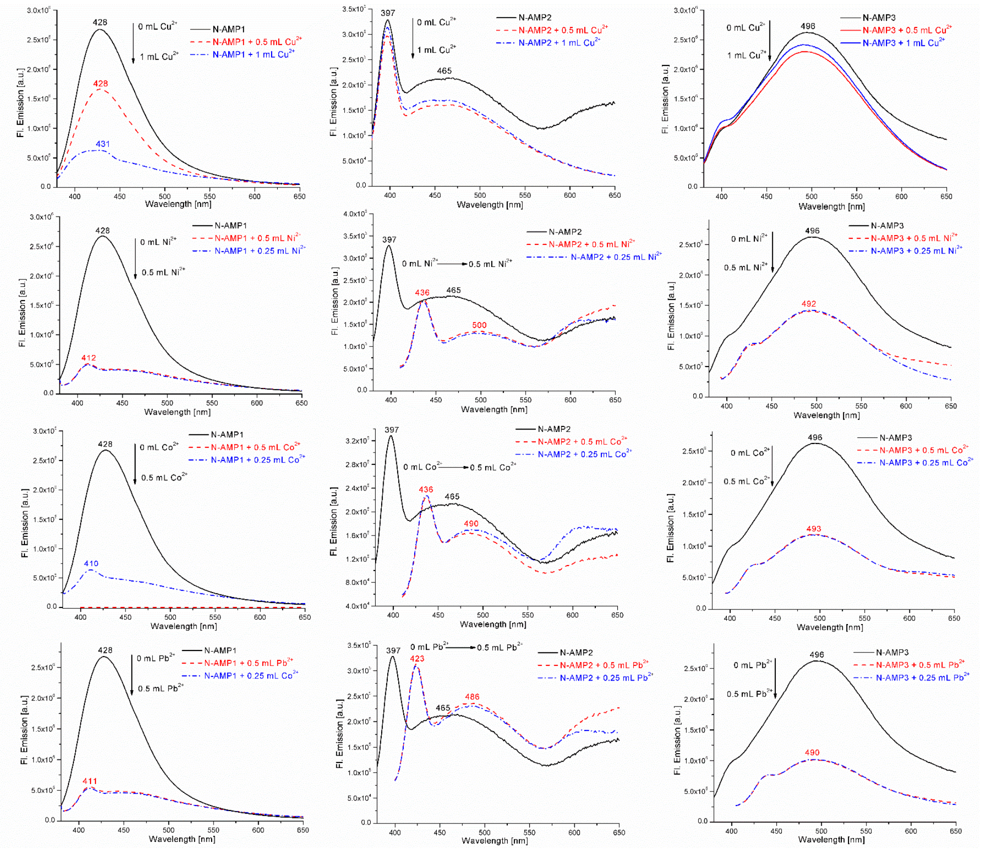

Figure S5. Fluorescence spectra of the N-AMP's (from left to right) and their metal complexes with *Cu(II)*, *Ni(II)*, *Co(II)*, *Pb(II)* (from top to bottom) with molar ratio metal : ligand = 1: 1 (the red lines) and 1:2 (the blue lines); the concentrations of the central ions: $3.238x10^{-5}$(Cu(II)), $8.52x10^{-5}$(Ni(II)), $8.89x10^{-5}$(Pb(II)) and $3.431x10^{-5}$(Co(II)) mol L^{-1} at 0.25ml from each ion.

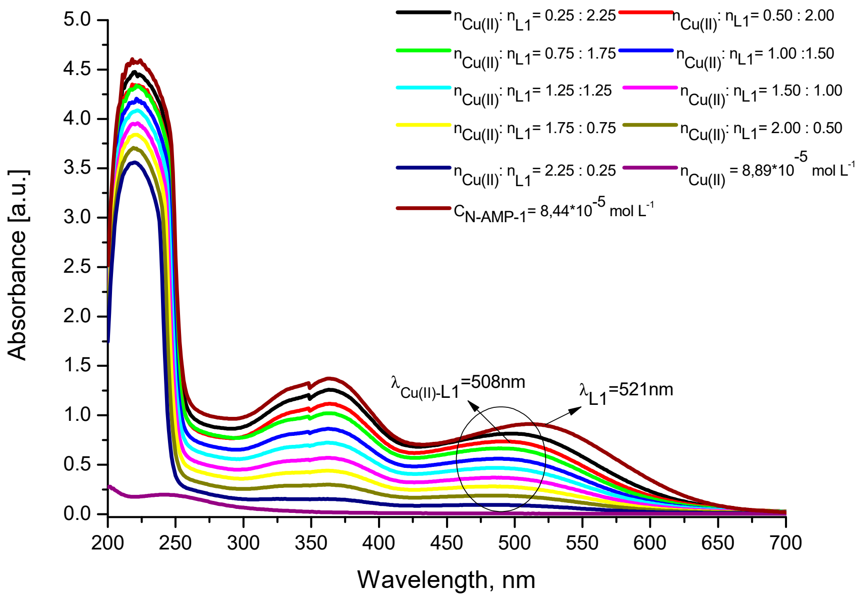

Figure S6. Uv-Vis absorption spectra of solutions containing Cu (II) and ligand: *N-AMP1*(*L1*) with different molar fractions of the two binding agents

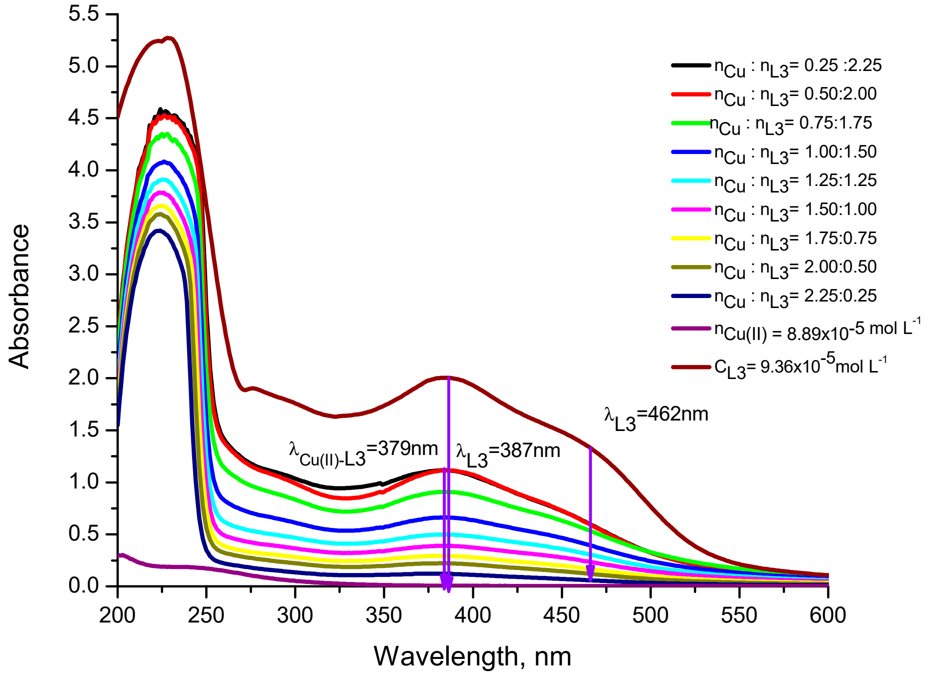

Figure S7. Uv-Vis absorption spectra of solutions containing Cu (II) and ligand: *N-AMP3*(*L3*) with different molar fractions of the two binding agents

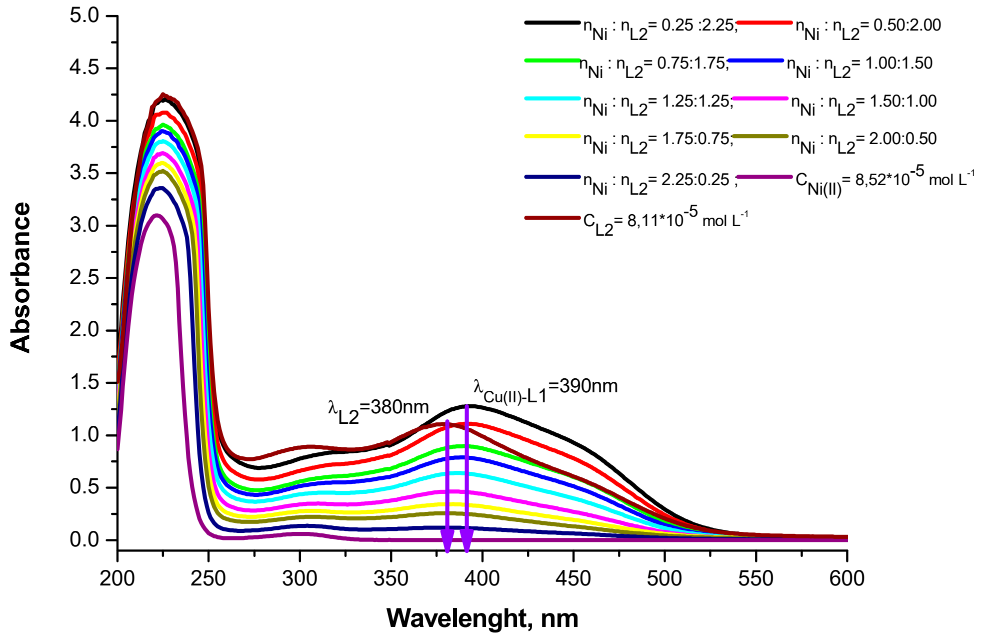

Figure S8. Uv-Vis absorption spectra of solutions containing Ni (II) and ligand: *N-AMP2*(*L2*) with different molar fractions of the two binding agents

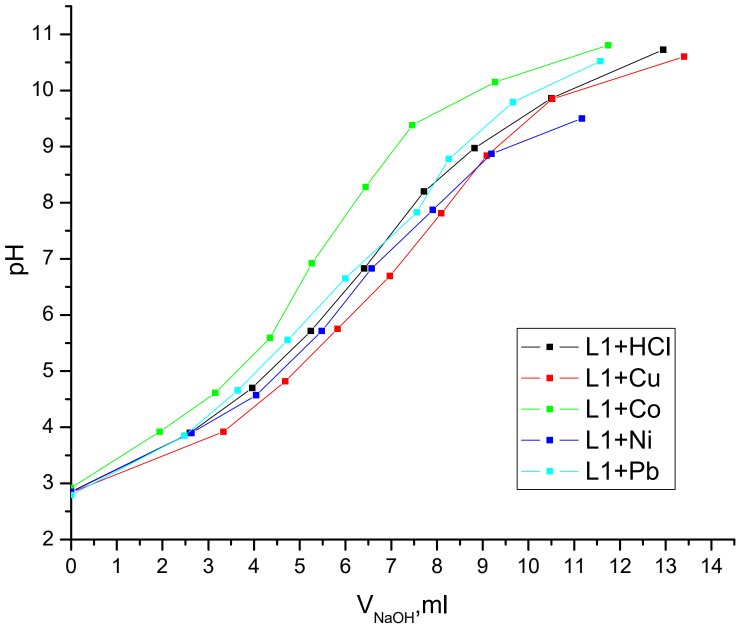

Figure S9. Plots of pH vs. V_{NaOH} obtained from potentiometric data at determination of the stability constants of metal-L1 complexes. The molar ration in the solution containing metal is:
metal ion : ligand = 1: 2.

Table S2. Values of constant formation and their logarithmic (logß) of obtained complexes of az-azomethine derivatives with metal ions at molar ratio metal : ligand = 1: 2

Compound	ß	logß
**Cu-L1**	$1.35x10^5$	5.13
**Cu-L2**	$1.74x10^5$	5.24
**Cu-L3**	$1.41x10^6$	6.15
**Ni-L1**	$4.17x10^4$	4.62
**Ni-L2**	$1.05x10^5$	5.02
**Ni-L3**	$1.45x10^5$	5.16
**Pb-L1**	$4.47x10^3$	3.65
**Pb-L2**	$1.62x10^4$	4.21
**Pb-L3**	$6.03x10^3$	3.78
**Co-L1**	$8.51x10^5$	5.93
**Co-L2**	$7.41x10^4$	4.87
**Co-L3**	$1.54x10^5$	5.19

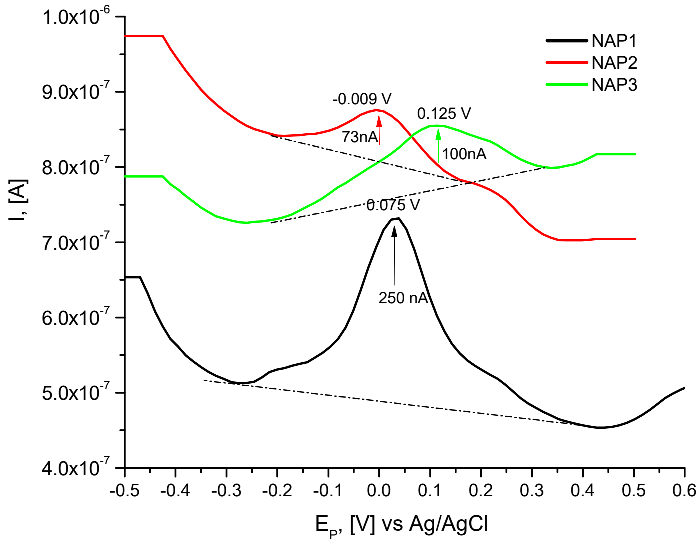

Figure S10. DP anodic voltammetry at Pt working electrode in 0.1 mol L^{-1} acetate buffer as a supporting electrolyte and equal concentrations of the ligands: $5.71x10^{-6}$ mol L^{-1}(L1), $5.69x10^{-6}$ mol L^{-1}(L2), $6.57x10^{-6}$ mol L^{-1}(L3)

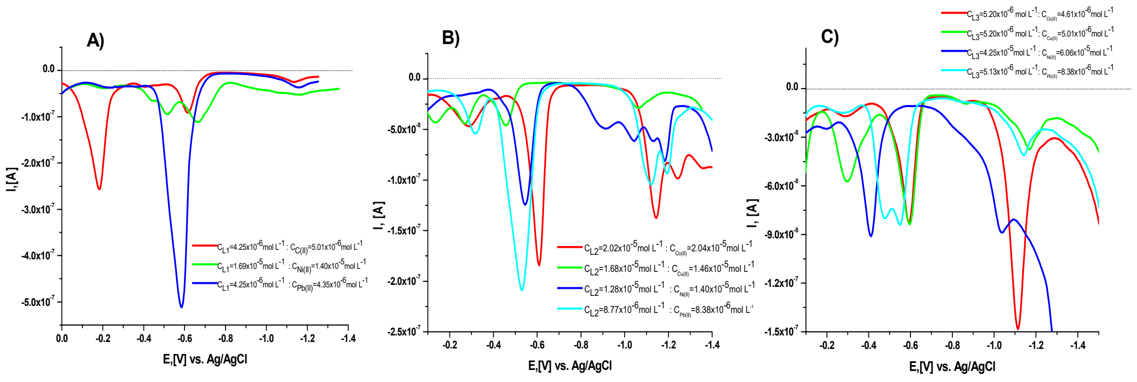

Figure S11. Differential pulse voltamperograms (cathodic) of azo-azomethine metal complex compounds in ammonium (pH 10.35, 0.1 mol L^{-1}) buffer solutions at HMDE working electrode and Ag/AgCl, KCl (3 mol L^{-1}) as reference electrode

Table S3. Values of potentials (**Ep, [V]**) and current intensities (**Ip, [A]**) of electroactive components (metal-ligand and unbound forms: metal and ligand) in ammonium (pH 10.35, 0.1 mol L^{-1}) and borate (pH 9.18, 0.1 mol L^{-1}) buffer solutions used as electrolyte medium at mercury working electrode in DPP mode.

Electroactive component	Electrolyte	Ep, [V]	Ip, [A]
**L1**	ABS/BBS	-0.621; -1.18/-0.646	-8.59x10^{-8}; 1.04x10^{-8}/-7.66x10^{-8}
**L2**	BBS/ABS	-0.581/-0.615; -1.25	-2.12x10^{-9} /-1.52x10^{-7}; -1.89x10^{-7}
**L3**	ABS	-0.58; -1.16	-8.38x10^{-8}; -3.1x10^{-8}
**Ni(II)**	ABS<br>BBS	-0.585; -0.871<br>-0.645; -0.829; -1.15	-5.78x10^{-8}; -4.92x10^{-8}<br>-6.81x10^{-8}; -1.49x10^{-8}; -1.11x10^{-8}
**Cu(II)**	ABS/BBS	-0.383; -0.591; -1.2/-0.182	-1.34x10^{-8}; -1.5x10^{-7}; -1.56x10^{-8}/-3.38x10^{-7}
**Pb(II)**	BBS/ABS	-0.518/-0.33; -0.556; -1.12	-5.63x10^{-7}/-1.08x10^{-8}; -1.25x10^{-7}; -9.25x10^{-8}
**Co(II)**	ABS/BBS	-0.585; -1.1/-1.84	-2.41x10^{-8}; -1.1x10^{-7}/-8.2x10^{-8}
**Cu(II)+L1**	BBS	-0.177; -0.615; -1.13	-2.24x10-^{7}; -7.42x10^{-8}; -1.38x10^{-8}
**Ni(II)+L1**	BBS	-0.2; -0.515; -0.64;<br>-0.872; -0.872; -1.15	-2.91x10^{-8}; -5.15x10^{-8}; -5.25x10^{-8}<br>-1.1x10^{-8}; -1.75x10^{-8}
**Co(II)+L1**	ABS<br>BBS	-0.222; -0.574<br>-0.636; -1.14; -0.182	-5.57x10^{-9}; -9.3x10^{-8}<br>-1.3x10^{-7}; -1.46x10^{-8}; -2.19x10^{-7}
**Pb(II)+L1**	BBS	-0.182; -0.581	-6.06x10^{-8}; -5.63x10^{-7}
**Cu(II)+L2**	ABS	-0,419; -0,556; -1,21	-1,9x10^{-7}; -3,56x10^{-7}; -1,11x10^{-7}
**N(II)+Li**	ABS<br>BBS	-0.562; -0.895; -1.06; -1.2<br>-0.621; -0.883, -1.08	-1.34x10^{-7}; -1.75x10^{-8}; -2.04x10^{-8}; -5.03x10^{-8};<br>-1.22x10^{-7}; -1.82x10^{-8}; -1.41x10^{-8}
**Co(II)+L2**	ABS	-0.288; -0.609; -1.14; -1.25	-3.07x10^{-8}; -1.73x10^{-7}; -1.14x10^{-8}<br>-1.95x10^{-8}
**Pb(II)-L2**	ABS	-0.324; -0.538; -1.12	-3.54x10^{-8}; -1.96x10^{-7};-9.25x10^{-8}
**Cu(II)-L3**	ABS	-0.3; -0.597; -1.17	-4.22x10^{-8}; -7.48x10^{-8}; -2.29x10^{-8}

**Ni(II)-L3**	ABS/BBS	-0.603; -0.913/-0.645	$-4.17x10^{-8}$; $-3.52x10^{-7}$/$-2.36x10^{-8}$
**Co(II)-L3**	ABS	-0.294; -0.591;-0.853; -1.12	$-5.82x10^{-9}$; $-7.59x10^{-8}$; $-1.66x10^{-9}$; - $1.29x10^{-7}$
**Pb(II)-L3**	ABS	-0.282; -0.55; -1.14	$-4.38x10^{-9}$; $-7.59x10^{-8}$; $-2.18x10^{-8}$
